# Effects of climate on West Nile Virus transmission risk used for public health decision-making in Quebec

**DOI:** 10.1186/1476-072X-6-40

**Published:** 2007-09-20

**Authors:** Salaheddine El Adlouni, Claudie Beaulieu, Taha BMJ Ouarda, Pierre L Gosselin, André Saint-Hilaire

**Affiliations:** 1 Hydro-Quebec/NSERC Chair in Statistical Hydrology, Canada Research Chair on the Estimation of Hydrological Variables, University of Quebec, INRS-ETE, 490, de la Couronne, Quebec (QC) G1K 9A9, CANADA; 2 Institut National de Santé Publique du Québec and Université Laval, 945, avenue Wolfe, Quebec (Quebec)G1V5B3, CANADA

## Abstract

**Background:**

In 2002, major human epidemics of West Nile Virus (WNV) were reported in five cities in the North East region of North America. The present analysis examines the climatic conditions that were conducive to the WNV epidemic, in order to provide information to public health managers who eventually must decide on the implementation of a preventive larvicide spraying program in Quebec, Canada. Two sets of variables, the first observed in the summer of 2002 and the second in the preceding winter were analysed to study their potential as explanatory variables for the emergence of the virus at epidemic levels.

**Results:**

Results show that the climatic conditions observed in the year 2002 have contributed to the emergence of the virus and can be observed once every forty years on average. The analysis has shown that the 2002 events observed in several North East North American cities are characterized by two main variables: the number of degree-days below -5°C in the winter (DD-5) and the number of degree-days greater than 25°C in the summer (DD25).

**Conclusion:**

In the context of a declining rate of human and aviary infection to WNV, this element contributed to the decision to suspend the use of preventive larvicides in the province of Quebec in 2006 and for the foreseeable future. The second part of this study indicates that it is very important to estimate the risk that extreme values can be observed simultaneously in the summer and in the winter preceding the appearance of the virus. The proposed models provide important information to public health officials, weeks before the appearance of the virus, and can therefore be useful to help prevent human epidemics.

## 1. Introduction

In 1999 the West Nile virus (WNV) was observed for the first time in North America. It is not known how the WNV was introduced in this region. The prevailing hypothesis is that migratory birds were the main vector [1] and the anomalous weather conditions may have provided the conditions for its amplification [2]. Studies conducted when the WNV occurred in the Nile delta of Egypt from 1952 to 1954, Romania in 1996, Russia in 1999 and USA in 1999 showed that weather conditions, mosquito behaviour and man made modifications of the environment can all impact on the transmission cycle of the WNV [3].

Shortly after the initial identification of the WNV in Uganda in 1937, researchers were able to highlight its similarity to two other viruses that were known to cause encephalitis: the St. Louis encephalitis (SLE) virus and the Japanese encephalitis (JE) virus [4]. The effect of summer temperatures and droughts on the mosquito extrinsic incubation period of the SLE, and thus on similar viruses, was noted [5]. In fact, periods of high temperatures seem to accelerate virus development, although very high temperatures are lethal to mosquitoes. This link between the WNV and climate was established by several other studies [6,7] which concluded that weather variability caused by climate instability contributed to the emergence of the WNV in North America. Especially, the climate experienced in the last six years seems to have helped the development of WNV among other epizootic viruses [8].

In 2002, important episodes of WNV were reported in five cities in the North East region of North America: Cleveland, Chicago, Detroit, New York and Toronto. The Greater Montreal area was not impacted at the same level than the aforementioned cities, in spite of the fact that it is located in the same region with a similar climate context. Only 20 cases and 3 deaths were reported and monitored in Montreal in 2002 [9] while several hundreds cases were reported in adjoining states and the cities mentioned above [10]. It was noticed that the climatic conditions of that specific year were particular. Given the decreasing and much lower level of activity of WNV in the province of Quebec since 2002 [9], the Quebec ministry of Health and Social Services (MSSS) requested in 2006 an expert opinion from its National Public Health Institute about the relevance of continuing the large scale use of larvicides as a preventive tool against WNV propagation. The present study was intended to contribute to that expert opinion and its objectives were to: (a) characterize the 2002 climatic conditions in Montreal and the five aforementioned cities and (b) evaluate the probability that such conditions will be observed in the future in the city of Montreal, Canada (located at 73°34' W and 45°31' N). The city of Montreal (about 1.5 M people) was selected as representative study area for its larger metropolitan area (about 3 M people).

## 2. Results

### 2.1 Univariate analysis

Table 1 presents the characteristics of the climatic variables observed in 2002. In the summer, the cumulated degree-days seemed particularly high in all six cities. However, the events of 2002 are not singular values: Grubbs-Beck tests [11] were carried out to confirm that degree-days for this period should not be considered as outliers. For the 2001–2002 winter periods, the minimal temperatures that were reached during the season are among the highest on record for all cities. The highest minimum temperatures were recorded in New York, Toronto and Montreal.

**Table 1:** Events of year 2002 and available observation periods for all considered variables

**Variable**	**Toronto**	**Cleveland**	**Detroit**	**Chicago (M)**	**Chicago (O)**	**NY (LGA)**	**NY (JFK)**	**Montreal**
**Tmax max (°C)**	35.4	35.0	36.1	36.7	35.6	36.7	37.2	35.6
**Tmin max (°C)**	24.6	23.9	23.3	26.7	25.6	27.8	26.7	24.0
**Tave max (°C)**	29.3	29.2	29.4	31.7	30.6	31.7	31.9	29.7
**Ptot (mm)**	513.9	514.4	488.4	621.0	661.9	772.2	760.2	416.3
**DD18 (°C)**	364.6	607.4	613.1	803.4	626.3	850.0	686.2	518.8
**DD20 (°C)**	208.1	388.6	398.3	565.6	413.3	593.9	442.5	324.4
**DD25 (°C)**	30.5	56.1	61.9	115.0	62.8	155.6	88.3	51.2
**Tave min (°C)**	-14.6	-10.3	-10.6	-13.3	-14.4	-3.1	-3.3	-8.5
**DD-5 (°C)**	338.6	748.9	650.6	764.4	623.9	1283.9	1082.2	548.6
**Available record**	1942–2002	1949–2002	1959–2002	1948–2002	1959–2002	1949–2002	1949–2002	1938–2002

Table 2 presents the statistical distributions selected for Montreal's climatic series and the non-exceedance probabilities for the 2002 events observed in the other stations. Rare events, which correspond to high non-exceedance probabilities, characterize the emergence of the 2002 WNV episode and these conditions can be considered as favourable for the outbreaks of the WNV. The exceedance probabilities are extremely high for the cumulated degree-days above -5°C observed in the winter (DD-5) and the cumulated degree-days above 25°C in the summer (DD25) of the year 2002 except for Toronto.

**Table 2:** No-exceedance probabilities of 2002 events using the Montreal model

**Variable**	**Distribution**	**Toronto**	**Cleveland**	**Detroit**	**Chicago (M)**	**Chicago (O)**	**NY (LGA)**	**NY (JFK)**	**Montreal**
**Tmax max**	Lognormal	0.739	0.665	0.844	0.903	0.765	0.903	0.943	0.772
**Tmin max**	Lognormal	0.969	0.926	0.865	0.999	0.992	1.000	0.999	0.935
**Tave max**	Lognormal	0.886	0.869	0.902	0.995	0.974	0.995	0.997	0.926
**Tave min**	GEV	0.906	0.992	0.991	0.951	0.913	1.000	1.000	0.998
**Ptot**	Lognormal	0.649	0.651	0.545	0.918	0.959	0.995	0.993	0.229
**DD18**	Lognormal	0.855	0.995	0.996	1.000	0.996	1.000	0.998	0.983
**DD20**	Lognormal	0.856	0.993	0.994	0.999	0.995	1.000	0.997	0.980
**DD25**	Gumbel	0.948	0.998	0.999	1.000	0.999	1.000	1.000	0.996
**DD-5**	Gumbel	0.679	0.999	0.998	1.000	0.996	1.000	1.000	0.987

### 2.2 Bivariate analysis

As shown in the previous section, the 2002 episodes observed in North America are characterized by two main temperature-related variables observed in the summer (variable DD25) and the winter (variable DD-5) which precedes the appearance of the human epidemics. It was shown in the previous section that the year 2002 events correspond to extreme values for both these variables. Further investigation of extreme values characteristics therefore requires a bivariate approach.

**Figure 1:**
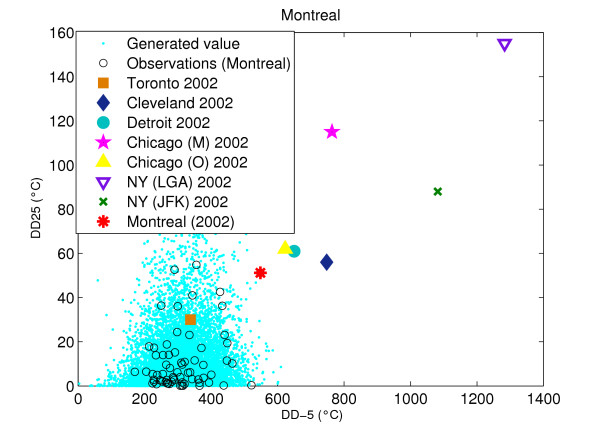
Bivariate model fitted to variables DD-5 and DD25 for Montreal data and observed 2002 events in all cities.

The Clayton copula (with *θ *= 0.1740) is selected and is used to define the bivariate distribution of DD-5 and DD25 for Montreal. Figure 1 presents the historic scatter plot of DD-5 and DD25 observed at Montreal, the 2002 events observed in all cities and the simulated values for Montreal. Simulated points are obtained from the theoretical bivariate model (using the marginal distributions, which is the Gumbel distribution for both variables, and the Clayton copula).

In order to truly compare the climatic conditions that led to a WNV outbreak in the four other cities with the conditions in Montreal, the same bivariate model was applied everywhere. The Montreal model was used as a reference. Table 3 illustrates the non-exceedance probabilities for the 2002 DD-5 and DD25 variables observed in all cities using the Montreal bivariate model. Results show that the 2002 events observed in all cities have low probabilities to be exceeded except for Toronto.

**Table 3:** Non-exceedance probabilities, using Montreal bivariate model, for 2002 events (DD-5, DD25) observed in all cities

	**Toronto**	**Cleveland**	**Detroit**	**Chicago (M)**	**Chicago (O)**	**NY (LGA)**	**NY (JFK)**	**Montreal**
*P*{*X *<*x*_2002_,*Y *<*y*_2002_}	0.56257	0.98374	0.98825	0.99963	0.98896	0.99997	0.99793	0.97648

Climatic conditions recorded in the year 2002 in all large cities considered in this study, except for NY (La Guardia (LGA) and Kennedy (JFK) stations) and Chicago (Midway (M) station), show significant probabilities to be exceeded (Figure 1 and Table 3). Thus, such events have a non-zero probability to be observed in Montreal. The estimated return periods for the observation of such events in Montreal are:

- Once every 60 years for Cleveland, Detroit, Chicago at station O'Hare (O), events;

- Once every 40 years for the Montreal event;

- Once every 2 years for Toronto.

## 3. Discussion

Climatic conditions are clearly not the unique factor linked to the emergence of the virus. Indeed, in 2002 the observed values of DD-5 and DD25 in Toronto did not correspond to high extremes although a high level of WNV human infections was observed in this region. Other factors such as the presence of migratory birds, humidity, proximity to other affected regions and previous exposure to the virus may also be responsible for the emergence of the virus even in the absence of such climatic conditions [12]. Indeed, more biological studies are recommended to explain why warm temperatures in the winter help the appearance of the virus.

The results of this study were nonetheless useful for decision-making for the Quebec Ministry of Health and Social Services (MSSS). From 2002 to 2005, the MSSS implemented thorough preventive measures and surveillance systems [13]. In the context of low and decreasing levels of activity of WNV in the province (45 proven cases over 4 years, with only one confirmed case in 2006), and given the low probability of recurrence of these favourable climatic conditions, the Quebec National Public Health Institute submitted two options for the future to the MSSS in early 2006 [14]. One option was continuing the current extensive program including the spraying of larvicides, while the other one proposed a much scaled-down program, keeping only human and mosquito surveillance, and the promotion of personal protection measures [14].

One other factor taken into account for final decision-making was the current knowledge on the efficiency of pesticide use in both preventive and epidemic situations. While its use seems efficient in epidemic situations [15,16], several important confounding factors were not factored in the epidemiologic analyses done so far [17] and currently no solid proof of pesticide efficiency against WNV in endemic or epidemic situations seems to be available in the opinion of the Institute [14]. Finally, economic considerations were also important in the decision-making process, as the costs of previous preventive programs were in the order of six million dollars per year, out of which 70% were allotted for pesticides (for larviciding), while the average costs for medical, hospital and other services related to the endemic situation were in the order of four hundred thousand dollars per year [14].

The MSSS has retained the low intervention option for 2006 and beyond, to be re-evaluated annually.

## 4. Conclusion

The present study investigated the principal climatic conditions which supported the emergence of the West Nile Virus in North America. First, the analysis has shown that the 2002 events observed in several North American cities are characterized by two main variables, DD-5 in the winter and DD25 in the summer. Indeed, recorded observations of these two variables (in all studied regions except for Toronto) correspond to extreme values with low probabilities of exceedance. The second part of this study investigated the joint effect of these two variables. It is very important to estimate the risk of simultaneous observation of extreme values in the summer and in the winter preceding the onset of the virus. Bivariate models allow to compute the probability that favourable conditions will be observed in the summer knowing that they have been observed during the winter. These models can provide important information to public health officials, weeks before the appearance of the virus, and can therefore be useful to help prevent human epidemics. In order to take into account our changing climate and the fact that historical data may become misleading for predictions, they can be also combined to long term meteorological forecasts outputs or climate scenarios such as the ones provided by Regional Climate Models (RCM) in order to predict or simulate future conditions that can lead to the occurrence of the virus.

## 5. Methods

### 5.1 Variable selection

In order to characterize climatic conditions of the 2002 WNV episode, several variables related to temperature and precipitation were analysed. Temperature and precipitation data were obtained from the *National Climatic Data Center *[18] for the cities of Chicago, Cleveland, Detroit and New York and from *Environment Canada *for Toronto and Montreal [19]. In Canada, the human episodes of WNV usually begin in July, reach their climax between mid-August and mid-September and decrease afterwards [8]. In 1999, when the WNV broke out for the first time in North America, a warm winter followed by a hot and dry summer were observed. Therefore, it seemed important to consider climatic conditions for the winter and summer seasons [2]. We thus considered two separate periods for the analysis of the climatic variables of interest: The first period goes from November until the end of March and serves to analyse the winter season preceding the appearance of the virus. The second period goes from April to the end of October.

Series of maximum, minimum temperatures and cumulated degree-days were extracted from daily temperature data for the period 1938–2002. All related variables are presented in Table 4. Series of total precipitation were also extracted from daily data. Since the time series of humidity that were available [18,19] were too short (between 1992 and 2002 only), this variable was not used in the present study. Table 4 describes all the variables considered in this work.

**Table 4:** Variables considered for the summer and winter periods

**Variable**	**Definition**	**Unit**	**Scale**
**Tmax max**	Maximum of daily maximum temperatures for the period of April 1^st ^to October 31^st^	°C	Year
**Tmin max**	Maximum of daily minimum temperatures for the period of April 1^st ^to October 31^st^	°C	Year
**Tave max**	Maximum of daily mean temperatures for the period of April 1^st ^to October 31^st^	°C	Year
**Tave min**	Minimum of daily mean temperature for the period of November 1^st ^to March 31^st^	°C	Year
**Ptot**	Cumulated total precipitations for the period of April 1^st ^to October 31^st^	mm	Year
**DD18**	Cumulated degree-days at threshold of 18°C for the period of April 1^st ^to October 31^st^	°C	Year
**DD20**	Cumulated degree-days at threshold of 20°C for the period of April 1^st ^to October 31^st^	°C	Year
**DD25**	Cumulated degree-days at threshold of 25°C for the period of April 1^st ^to October 31^st^	°C	Year
**DD-5**	Cumulated degree-days at threshold of -5°C for the period of November 1^st ^to March 31^st^	°C	Year

### 5.2 Univariate statistical analysis

The main objective of this preliminary analysis was to characterize the 2002 WNV episodes through related climatic variables. First, an exploratory analysis was made in order to characterise the climatic conditions that correspond to the 2002 WNV episodes.

Frequency analysis was used to evaluate the risk of occurrence in Montreal of the climatic conditions that were favourable to the development of an epidemic of WNV in the other five cities. Statistical distributions (Normal, Lognormal, Gumbel, Generalized Extreme value, Gamma) were fitted to the considered climatic variables for Montreal and probabilities of exceedance were evaluated. The selection of the most adequate distributions was made according to the Akaïke information criterion [20] and the Bayesian information criterion [21]. Thus, the risk that climatic conditions conducive to a WNV epidemic can be observed in Montreal was evaluated.

### 5.3 Bivariate analysis

The main objective of the approach presented in this section, is to identify important combinations of two factors and to evaluate the risk associated with these combinations. Indeed, as noted in the introduction, warm winters followed by hot, dry summers seem to promote the transmission of the WNV [2]. This assumption is examined in this section through joint non-exceedance probabilities of two climatic variables associated to the 2002 episode.

The joint effect of climatic variables that are conducive to the emergence of the WNV episodes was modeled using multivariate statistical methods. As the use of a multivariate normal distribution is not appropriate to model extreme values in this situation [22], we used a more general approach based on copulas [23]. A copula is a distribution function whose margins are uniform. Copulas are useful because they allow the modelling of the dependency relationships among random variables independently of their marginal distributions. This is important because copulas can handle dependency between random variables without the limitations of the more familiar dependency measure, the linear correlation coefficient.

To briefly outline the concept of a copula function, define the bivariate joint distribution *H *of random variables X and Y, in terms of copula *C *and marginal *F*_1 _and *F*_2 _such that: *H*(*X*, *Y*) = *C*(*F*_1_(*X*), *F*_2_(*Y*)). Variables X and Y should be continuous to get uniform distribution on the unit interval by putting *U *= *F*_1_(*X*) and *V *= *F*_2_(*Y*). Then copula *C*(*U*, *V*) captures the dependence structure of *H*(*X*, *Y*). The Archimedean copula family contains a large variety of copulas that represent different dependence structures. In this study we use three copulas (Clayton, Frank and Gumbel) belonging to the Archimedean copula family. Table 5 summarizes the most important one-parameter Archimedean copulas and their generator functions. Note that the copula parameter can be estimated using the maximum likelihood method or using the moment method through the relation between the parameter and dependence measure such as Kendall's *τ *or Spearman's *ρ*. For samples *X*_1_, ..., *X*_*n *_and *Y*_1_, ..., *Y*_*n *_the Kendall's *τ *can be estimated by: τ(X,Y)=(n2)−1∑1≤t<s≤nsign((Xt−Xs)(Yt−Ys))
 MathType@MTEF@5@5@+=feaafiart1ev1aaatCvAUfKttLearuWrP9MDH5MBPbIqV92AaeXatLxBI9gBaebbnrfifHhDYfgasaacH8akY=wiFfYdH8Gipec8Eeeu0xXdbba9frFj0=OqFfea0dXdd9vqai=hGuQ8kuc9pgc9s8qqaq=dirpe0xb9q8qiLsFr0=vr0=vr0dc8meaabaqaciaacaGaaeqabaqabeGadaaakeaaiiGacqWFepaDcqGGOaakcqWGybawcqGGSaalcqWGzbqwcqGGPaqkcqGH9aqpdaqadaqaauaabeqaceaaaeaacqWGUbGBaeaacqaIYaGmaaaacaGLOaGaayzkaaWaaWbaaSqabeaacqGHsislcqaIXaqmaaGcdaaeqbqaaiabdohaZjabdMgaPjabdEgaNjabd6gaUnaabmaabaWaaeWaaeaacqWGybawdaWgaaWcbaGaemiDaqhabeaakiabgkHiTiabdIfaynaaBaaaleaacqWGZbWCaeqaaaGccaGLOaGaayzkaaWaaeWaaeaacqWGzbqwdaWgaaWcbaGaemiDaqhabeaakiabgkHiTiabdMfaznaaBaaaleaacqWGZbWCaeqaaaGccaGLOaGaayzkaaaacaGLOaGaayzkaaaaleaacqaIXaqmcqGHKjYOcqWG0baDcqGH8aapcqWGZbWCcqGHKjYOcqWGUbGBaeqaniabggHiLdaaaa@5DC6@.

**Table 5:** Archimedean copulas used in this study with their generators

Family of copulas	Generator *φ *(*t*)	Parameter *θ*	Bivariate Copula *C*_*φ *_(*u*, *v*)
Independent	-ln(*t*)		*uv*
Gumbel	(-ln(*t*))^*θ*^	*θ *≥ 1	e−[(−ln⁡u)θ+(−ln⁡v)θ]1/θ MathType@MTEF@5@5@+=feaafiart1ev1aaatCvAUfKttLearuWrP9MDH5MBPbIqV92AaeXatLxBI9gBaebbnrfifHhDYfgasaacH8akY=wiFfYdH8Gipec8Eeeu0xXdbba9frFj0=OqFfea0dXdd9vqai=hGuQ8kuc9pgc9s8qqaq=dirpe0xb9q8qiLsFr0=vr0=vr0dc8meaabaqaciaacaGaaeqabaqabeGadaaakeaacqWGLbqzdaahaaWcbeqaaiabgkHiTmaadmaabaGaeiikaGIaeyOeI0IagiiBaWMaeiOBa4MaemyDauNaeiykaKYaaWbaaWqabeaaiiGacqWF4oqCaaWccqGHRaWkcqGGOaakcqGHsislcyGGSbaBcqGGUbGBcqWG2bGDcqGGPaqkdaahaaadbeqaaiab=H7aXbaaaSGaay5waiaaw2faamaaCaaameqabaGaeGymaeJaei4la8Iae8hUdehaaaaaaaa@4734@
Clayton	*t*^-*θ *^-1	*θ *> 0	(*u*^-*θ *^+ *v*^-*θ *^-1)^-1/*θ*^
Frank	−ln⁡(e−θt−1e−θ−1) MathType@MTEF@5@5@+=feaafiart1ev1aaatCvAUfKttLearuWrP9MDH5MBPbIqV92AaeXatLxBI9gBaebbnrfifHhDYfgasaacH8akY=wiFfYdH8Gipec8Eeeu0xXdbba9frFj0=OqFfea0dXdd9vqai=hGuQ8kuc9pgc9s8qqaq=dirpe0xb9q8qiLsFr0=vr0=vr0dc8meaabaqaciaacaGaaeqabaqabeGadaaakeaacqGHsislcyGGSbaBcqGGUbGBdaqadaqaamaalaaabaGaemyzau2aaWbaaSqabeaacqGHsisliiGacqWF4oqCcqWG0baDaaGccqGHsislcqaIXaqmaeaacqWGLbqzdaahaaWcbeqaaiabgkHiTiab=H7aXbaakiabgkHiTiabigdaXaaaaiaawIcacaGLPaaaaaa@3F7F@	*θ *∈ ℝ	−1θln⁡(1+(e−θu−1)(e−θv−1)(e−θ−1)) MathType@MTEF@5@5@+=feaafiart1ev1aaatCvAUfKttLearuWrP9MDH5MBPbIqV92AaeXatLxBI9gBaebbnrfifHhDYfgasaacH8akY=wiFfYdH8Gipec8Eeeu0xXdbba9frFj0=OqFfea0dXdd9vqai=hGuQ8kuc9pgc9s8qqaq=dirpe0xb9q8qiLsFr0=vr0=vr0dc8meaabaqaciaacaGaaeqabaqabeGadaaakeaacqGHsisldaWcaaqaaiabigdaXaqaaGGaciab=H7aXbaacyGGSbaBcqGGUbGBdaqadaqaaiabigdaXiabgUcaRmaalaaabaWaaeWaaeaacqWGLbqzdaahaaWcbeqaaiabgkHiTiab=H7aXjabdwha1baakiabgkHiTiabigdaXaGaayjkaiaawMcaamaabmaabaGaemyzau2aaWbaaSqabeaacqGHsislcqWF4oqCcqWG2bGDaaGccqGHsislcqaIXaqmaiaawIcacaGLPaaaaeaadaqadaqaaiabdwgaLnaaCaaaleqabaGaeyOeI0Iae8hUdehaaOGaeyOeI0IaeGymaedacaGLOaGaayzkaaaaaaGaayjkaiaawMcaaaaa@5019@

Archimedean copulas are very easy to implement because of the closed expression of the dependence parameter as a function of Kendall's *τ*. Table 6 shows particular closed forms of this relation. An estimator of the copula parameter can be obtained using the empirical estimate of Kendall's *τ*.

**Table 6:** Kendall's measure of association and Archimedean copulas

Family of copulas	Independent	Gumbel	Clayton	Frank
Kendall's *τ*	0	θ−1θ MathType@MTEF@5@5@+=feaafiart1ev1aaatCvAUfKttLearuWrP9MDH5MBPbIqV92AaeXatLxBI9gBaebbnrfifHhDYfgasaacH8akY=wiFfYdH8Gipec8Eeeu0xXdbba9frFj0=OqFfea0dXdd9vqai=hGuQ8kuc9pgc9s8qqaq=dirpe0xb9q8qiLsFr0=vr0=vr0dc8meaabaqaciaacaGaaeqabaqabeGadaaakeaadaWcaaqaaGGaciab=H7aXjabgkHiTiabigdaXaqaaiab=H7aXbaaaaa@3207@	θθ+2 MathType@MTEF@5@5@+=feaafiart1ev1aaatCvAUfKttLearuWrP9MDH5MBPbIqV92AaeXatLxBI9gBaebbnrfifHhDYfgasaacH8akY=wiFfYdH8Gipec8Eeeu0xXdbba9frFj0=OqFfea0dXdd9vqai=hGuQ8kuc9pgc9s8qqaq=dirpe0xb9q8qiLsFr0=vr0=vr0dc8meaabaqaciaacaGaaeqabaqabeGadaaakeaadaWcaaqaaGGaciab=H7aXbqaaiab=H7aXjabgUcaRiabikdaYaaaaaa@31FE@	1−4θ{1−D1(θ)} MathType@MTEF@5@5@+=feaafiart1ev1aaatCvAUfKttLearuWrP9MDH5MBPbIqV92AaeXatLxBI9gBaebbnrfifHhDYfgasaacH8akY=wiFfYdH8Gipec8Eeeu0xXdbba9frFj0=OqFfea0dXdd9vqai=hGuQ8kuc9pgc9s8qqaq=dirpe0xb9q8qiLsFr0=vr0=vr0dc8meaabaqaciaacaGaaeqabaqabeGadaaakeaacqaIXaqmcqGHsisldaWcaaqaaiabisda0aqaaGGaciab=H7aXbaadaGadeqaaiabigdaXiabgkHiTiabdseaenaaBaaaleaacqaIXaqmaeqaaOWaaeWaaeaacqWF4oqCaiaawIcacaGLPaaaaiaawUhacaGL9baaaaa@3ACC@

Note: Dk(x)=kxk∫0xtket−1
 MathType@MTEF@5@5@+=feaafiart1ev1aaatCvAUfKttLearuWrP9MDH5MBPbIqV92AaeXatLxBI9gBaebbnrfifHhDYfgasaacH8akY=wiFfYdH8Gipec8Eeeu0xXdbba9frFj0=OqFfea0dXdd9vqai=hGuQ8kuc9pgc9s8qqaq=dirpe0xb9q8qiLsFr0=vr0=vr0dc8meaabaqaciaacaGaaeqabaqabeGadaaakeaacqWGebardaWgaaWcbaGaem4AaSgabeaakmaabmaabaGaemiEaGhacaGLOaGaayzkaaGaeyypa0ZaaSaaaeaacqWGRbWAaeaacqWG4baEdaahaaWcbeqaaiabdUgaRbaaaaGcdaWdXaqaamaalaaabaGaemiDaq3aaWbaaSqabeaacqWGRbWAaaaakeaacqWGLbqzdaahaaWcbeqaaiabdsha0baakiabgkHiTiabigdaXaaaaSqaaiabicdaWaqaaiabdIha4bqdcqGHRiI8aaaa@4457@ is called "Debye" function

Archimedean copulas are used here to represent the bivariate distribution of combinations of factors that seem to contribute to the emergence of the WNV. To estimate the joint risk and the probability that such events occur simultaneously, we used the bivariate distribution of combinations of the above mentioned factors. Marginal distributions fitted by univariate analysis are combined to the Archimedean copula which gives the best fit in order to define the joint distribution. Copula selection is done through the K-test [23,24].

## Authors' contributions

All authors contributed to the writing of the paper. SEA, CB and TO conducted the development of the univariate and bivariate statistical models. PG helped provide access to the WNV data and contributed to the interpretation of results. ASH contributed to the selection of the meteorological variables and to the analysis interpretation. All authors read and approved the final version of the manuscript.
